# Influence of Freeze–Thaw Cycles on the Mechanical Properties of Highly Rubberised Asphalt Mixtures Made with Warm and Cold Asphalt Binders

**DOI:** 10.3390/ma15072701

**Published:** 2022-04-06

**Authors:** Christina Makoundou, Cesare Sangiorgi

**Affiliations:** Department of Civil, Chemical, Environmental and Materials Engineering, University of Bologna, 40131 Bologna, Italy; cesare.sangiorgi4@unibo.it

**Keywords:** rubberised asphalt, crumb rubber, cold mix, warm mix, bitumen emulsion, polymer-modified bitumen, freeze-thaw, durability, particle loss, leaching

## Abstract

The present study has been developed to investigate the effect of freeze and thaw (F–T) cycles on the characteristics of highly rubberised asphalt materials to be used as impact-absorbing pavement (IAP) in urban road infrastructures. The tested samples were produced in the laboratory following the dry process incorporation. Two main types of crumb rubber particles in the range of 0–4 mm were used. Moreover, two types of binders, one warm and one cold, were utilised to prove the feasibility of cold-produced admixtures. The temperature range of the F–T procedure was comprised between −18 ± 2 °C (dry freezing), and 4 ± 2 °C (in water), and the cycles were repeated, on the samples, 10 times. At 0, 1, 5, and 10 cycles, the samples were tested with non-destructive and destructive testing methods, including air voids content, ITSM, ITS, and Cantabro loss. The waters of the thawing period were collected, and the pH, electric conductivity, and particle loss were measured. A consequent change in mechanical behaviour has been recorded between warm and cold produced samples. However, the tests found that the F–T cycles had limited influence on the deterioration of the highly rubberised samples. The loss of particles in the thaw waters were identified as being potentially caused by the temperature stresses. The research suggested various ways to optimise the material to enhance the cold-produced layer mechanical performances, aiming at a fume and smell-free industrialised solution and reducing the potential leaching and particle losses.

## 1. Introduction and Objective

Asphalt pavements are common materials for constructing transportation infrastructures, and they have been developed and optimised for many years [1]. While designing or improving the recipe of bituminous paving material, the mechanical loads applied by traffic and the climate actions, resulting in stresses and strains, moisture damage, temperature changes, and ageing, have an important role. These two main elements are always responsible for most pavement deterioration. Nevertheless, temperature variations and freeze–thaw cycles are known to be the main factors responsible for thermal fatigue and potholes because of the induction of water-caused distresses, which are the main causes of pavement failures in rigid climate regions [2,3].

In the past years, the evaluation of the weather's influence on asphalt materials, especially with cold temperatures, has been investigated in many studies [4,5,6,7,8,9,10]. The development of a correlation between the pavement temperature conditions and the mix design of the bituminous material in the targeted cold region was proven to be an important aspect of the mixture optimisation. 

The research topic is more developed in regions with a relatively wide temperature range characterising climate, and where the pavement is usually subject to freeze–thaw (F–T) cycles. For instance, a study from El-Hakim et al. [2] presented a statistical assessment of the impact of F–T cycles on the deterioration of the mechanical properties of asphalt mixes in Canada. Other groups studied the effects of F–T cycles on the performances of asphalt mixtures in the cold regions of China and Iran [11,12,13,14], while in Sweden Lövqvist et al. implemented a model to show the effects of different parameters on the materials (the number of F–T cycles, the gradation of the microstructure, and the freezing time) thanks to computer-assisted F–T simulations [15]. The work done on F–T cycles can be easily applied to other cold regions as a conditioning procedure reproducing the climate evolution and the related F–T scenarios.

As far as rubberised pavements are concerned, a limited number of studies can be found on the F–T of paving materials containing rubber, even less on highly rubberised materials, despite the environmental advantages connected with the use of recycled end-of-life tyres (ELTs) rubber [16]. A study conducted by Guo et al. demonstrates the correlation between the use of crumb rubber in a bituminous mixture and the reduction of the anti-fatigue performances and life of the specimen after F–T cycles [17]. However, some major limitations of using rubber in asphalt concretes are the swelling and expansion that has a role in the degradation of the material [18]. The number of voids can turn the material more prone to deterioration caused by water infiltration. Moreover, the rubber tends to absorb the binder, allowing the adhesion and cohesion between the aggregates, partly allowing water ingress at interfaces and consequent damages [19]. However, in the parallel field of cementitious materials, a study conducted by Richardson et al. stated that crumb rubber particles smaller than 0.5 mm are optimal to provide freezing protection in rubberised concrete [20].

A similar observation has also been made with reference to the use of cold emulsified asphalt binder mixtures for chip-sealing purposes. You et al. focused on the durability characteristics of asphalt emulsion-based chip-seals and the effect of the asphalt aggregate combination at cold temperatures when exposed to multiple F–T cycles [21]. 

In the light of the above, this paper presents the results of geometrical, volumetric, and mechanical characterisation, carried out on highly-rubberised samples made with crumb rubber, warm and cold asphalt binders after freeze and thaw (F–T) cycles applied following the ASTM C666/C666M standard (Procedure B) [22]. The main research objective is to evaluate the resistance of the developed rubberised asphalt mixture—intended to be used as an impact-absorbing pavement (IAP) [23]—to repeated cycles of F–T. No comparisons with ordinary and traditional asphalt are presented because of the vast disparity in performance and application intentions, especially regarding the strength and stiffness of the developed rubberised asphalt.

## 2. Materials and Methods

### 2.1. Raw Materials

The tested mixtures are made with two types of recycled rubber (with different size distributions from 0 to 4 mm) and a Polymer-modified Binder (PmB) or a Polymer-modified bituminous Emulsion (PmE). The adopted mixture design resulted from previous studies developed to define a viable and consistent formulation [23].

Ambient shredded rubber from two different suppliers, trough the collaboration of the Swedish Tyre Recycling Association,) (from Northern Europe) was used to produce the rubberised samples. The rubber size distribution ranged between 0 and 4 mm, but the various products have different sieving curves. The rubber from both sources alternatively substitutes a portion of the mineral aggregates in the designed mixtures according to different specific volumetric recipes. Rubber characteristics are shown in Table A1 and the sieving analysis in Table A2.

Limestone aggregates ranging from 4 to 14 mm and limestone filler (below 0.063 mm) were used to create the bituminous mastic and the lithic structure of the designed pavement. Intermediate-size aggregates (0–4 mm) were partially substituted with rubber of the same size to provide the material with the required elastic and impact-absorbing properties. The mineral aggregates' characteristics are listed in Table A3 and Table A4.

Two types of binders from the same supplier were used in this study. The first one is a warm styrene-butadiene-styrene (SBS) Polymer-modified Bitumen (PmB) that was purposely designed to be used with rubber powders, and the second one is an SBS Polymer-modified Emulsion (PmE) containing 67–69% of residual bitumen. The binders’ properties are given in Table A5 and Table A6.

### 2.2. Highly-Rubberised Asphalt Concrete Samples’ Production

**Figure 1:**
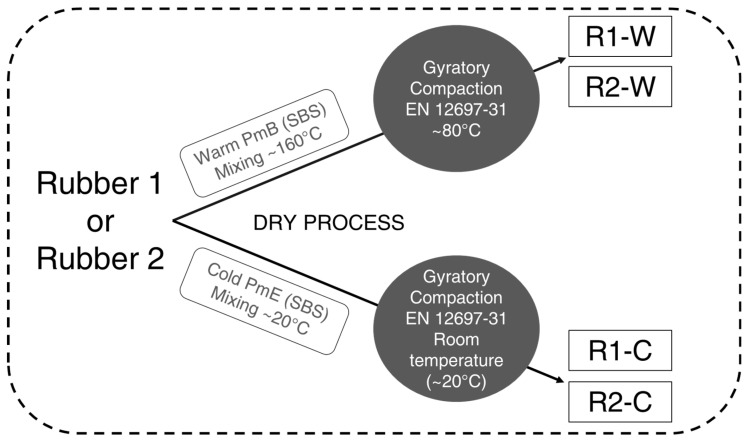
Rubberised asphalt production procedure.

As illustrated in Figure 1, two different mixing and compaction procedures, corresponding to the variation of the binder type and the mixing and compaction temperatures were adopted. In both cases, the dry process was used to incorporate the rubber into the mixes.

In the case of the warm mix asphalt, the aggregates and the bitumen were heated at 160 °C in the oven before starting the procedure. At the beginning of the mixing process, the aggregates were added and homogenised for a few minutes in the mixer. Secondly, the bitumen and the filler were incorporated. The bituminous mixture was then mixed for five minutes. Finally, the crumb rubber mix was added, and the final mixture was mixed until homogenisation. After this process, the obtained mixture was divided into portions required to obtain a 100 mm diameter and approximatively 40 mm thickness sample (approx. 600 g). The trays with the mixture were kept for a short period at a constant temperature corresponding to the adopted compaction one, i.e., 80 °C. 

As for the production of the cold mix asphalt concrete, the aggregates, filler, and rubber particles are mixed and well homogenised before the addition of the emulsion. Using the mixture’s organoleptic assessment preconised for the mixtures made with emulsions (EN 12697-55) [24], the mixing time was identified in less than five minutes. At the end of the mixing time, the mixture had a wet light-brown colour corresponding to that of an unbroken emulsion. This mixture was then weighted and divided into portions before the compaction at room temperature (approx. 20 °C). 

The gyratory compactor (EN 1269-31) [22] was used for warm and cold mixtures. Each sample was compacted for 80 cycles. Twelve samples have been produced for each mix, and, independently of the production procedure, all samples incorporated the same amount of rubber, aggregates, and bitumen as listed in Table 1.

**Figure 2:**
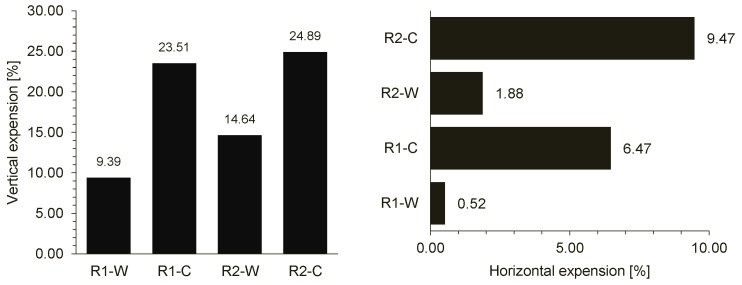
Vertical and horizontal expansion of the samples.

Additionally, the specimens’ expansion from the original dimensions was measured right before and after one week from their compaction. The final expansion percentage is given in Figure 2. The cold-made samples expanded more than the warm-made samples, and the Rubber 1 samples expanded less than the Rubber 2 samples.

### 2.3. Freeze and Thaw Conditioning Procedure

In addition to the effect of traffic loads, pavement deterioration can also be caused by cyclic climate actions. In recent years, thermal cracks and low temperature distresses have become a key concern for asphalt pavements in cold regions. In the laboratory, as in the real case, during the F–T cycles, the air temperature recurrently changes from positive to negative, and the layer is subject to repeated thermal stresses and related moisture effects. The compressive strength and the resilient modulus of the asphalt mix usually decrease when standard asphalt concrete is subjected to F–T cycles. Therefore, in this study, F–T cycles were also applied to the developed rubberised asphalt concrete to characterise and evaluate its behaviour in harsh conditions. 

**Figure 3:**
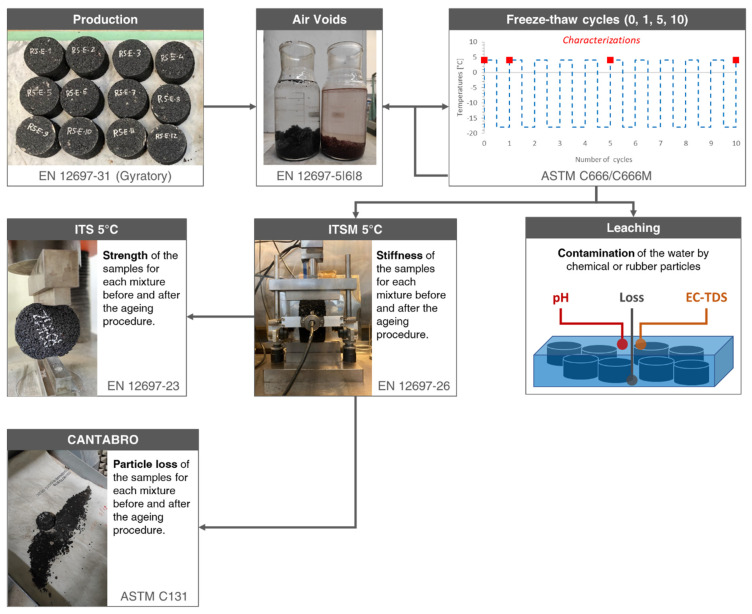
Summary of the experimental programme for each F–T cycle.

The samples were produced to carry out an F–T ageing procedure to analyse the cyclic temperatures, ice, and moisture effect on the degradation of rubberised samples. A sufficient amount of time of approximately 5 days was allowed before applying the cycles to provide complete curing for all samples. After a defined number of cycles (0, 1, 5, and 10), volumetric, mechanical, and durability tests were conducted as schematised in Figure 3.

Following ASTM C666/C666-M [22], after the curing period and before starting the F–T cycles, each sample was conditioned for 48 h in water at 23 ± 2 °C (room temperature). After the conditioning phase, the actual F–T cycles were started with a freezing phase set to reach −18 ± 2 °C and maintained for three hours (180 min), followed by the thawing phase carried out in cold water to reach +4 ± 2 °C and maintained for one hour and a half (90 min). 

All samples of the control group (0 cycles) were tested following the ITSM procedure (EN 12697-26) [25]. Successively, some samples were destructively tested utilising the ITS (EN 12697-23) [26], while others underwent the Cantabro test (ASTM C131) [27]. This characterisation procedure was repeated on twin samples at 1 cycle, 5 cycles, and 10 cycles of F–T, as shown in Figure 2. Finally, to obtain an outcome of the leaching potential of the samples when in contact with water, the pH and EC-TDS values of the thaw waters were analysed at the end of the cycles. The protocol of each method is detailed in the following sections, and the complete experimental programme is schematised in Figure 3. 

### 2.4. Characterisation

After being produced and conditioned, the samples were characterised prior to the F–T procedure and during the treatment protocol at 1, 5, and 10 F–T cycles. Consequently, the air voids’ content and densities [21,22,23], the Indirect Tensile Stiffness Modulus (ISTM) [25], the Indirect Tensile Strength (ITS) [26], and the Cantabro loss (CL) [27] results were recorded after each set of cycles. In addition, the pH and electric conductivity were also measured for each thaw water to investigate the potential leaching of the rubberised samples after prolonged contact with water in cold conditions. 

#### 2.4.1. Densities and Air Voids’ Content

The samples’ bulk and maximum densities were calculated following the EN 12697-6 [28] and EN 12697-5 [29] standards. After obtaining the densities values, the air voids’ content was calculated as specified in the EN 12697-8 standard [30] using the previous parameters.

#### 2.4.2. Indirect Tensile Stiffness Modulus

The stiffness of the asphalt mixture has always been considered one of the main indicators of the material’s mechanical properties at various temperatures, especially the extreme ones. In the case of highly rubberised mixtures, stiffness reveals how the rubber content influences the overall mix behaviour under dynamic loading. Thus, the ITSM was used to investigate the stiffness modulus of the rubberised mix asphalt at low temperatures, i.e., when surface layers are usually stiffer and prone to thermal cracking. The analysis was carried out following the EN 12697-26 [25] standards. It was chosen to adopt a 5 °C test temperature as an average low temperature enabling consistent testing and as an assessment of the stiffness in cold weather for a material conceived to be working at those temperatures. All samples have undergone 4-hour conditioning at 5 °C before being tested in the pre-cooled ITSM chamber. 

#### 2.4.3. Indirect Tensile Strength

Like ITSM, the *ITS* is an indicator of the material’s mechanical properties, particularly the maximum load applied before indirect tensile failure. The EN 12697-23 standard [22] has been used for its assessment. The cylindrical specimen is loaded diametrically with a constant displacement rate until failure. The indirect tensile strength is calculated according to the following equation:

(1) $$ITS\;\left[\mathrm{MPa}\right]=\frac{2\times P}{\pi \times D\times H}$$

*ITS* is the Indirect Tensile Strength, expressed in MPa; *P* is the peak load, expressed in N; *D* is the diameter of the specimen, expressed in mm and *H* is the height of the specimen, expressed in mm.

#### 2.4.4. Cantabro Loss

The Cantabro test procedure measures the cohesive properties of compacted specimens using the Los Angeles Abrasion Machine. The percentage of weight loss (Cantabro loss) indicates the material durability and relates to the quantity and quality of the asphalt binder and mixture compaction. The Los Angeles machine is set to 30–33 revolutions per minute for 300 revolutions. After 300 revolutions, the loose mix (if applicable) is discarded, as shown in Figure 3, and the test specimen is weighted. ASTM C131 standard [23] was followed to carry out this test.

(2) $$CL\;\left[\%\right]=\frac{W_{ini}\times W_{fin}}{W_{ini}}\times 100$$

*CL* is the Cantabro Loss [%]; *W*_*ini*_ is the initial weight of the test specimen [g] and *W*_*fin*_ is the weight of the test specimen after the test completion [g].

#### 2.4.5. Measures of Particle Loss, pH, and Electric Conductivity-Total Dissolved Solid 

The leaching behaviour of the specimens was evaluated by analysing the thaw waters with a basic pH and EC-TDS meter. It was conducted to record the changes over time due to potentially released chemicals coming from the leaching of the asphalt specimen immersed in water. This tracking can show how leaching evolves.

The water was poured into the thawing tray, and the two parameters were measured at t = 0. Thus, samples (approximately 12 in each thaw tray) were immersed in water. Ten minutes later, the measurement was repeated. Additional pH and EC-TDS values were collected: one after the conditioning period of 48 h at room temperature before the start of the F–T process and others at the end of the F–T cycles number 1, 5, and 10.

In parallel with the leaching evaluation, the particles lost in the thawing water were collected, dried, and weighted to obtain a cumulative percentage of loss compared to the total weight of samples initially submerged in the tray. This measurement was made to control the potential particles loss to minimise micro-particle release in the environment.

**Figure 4:**
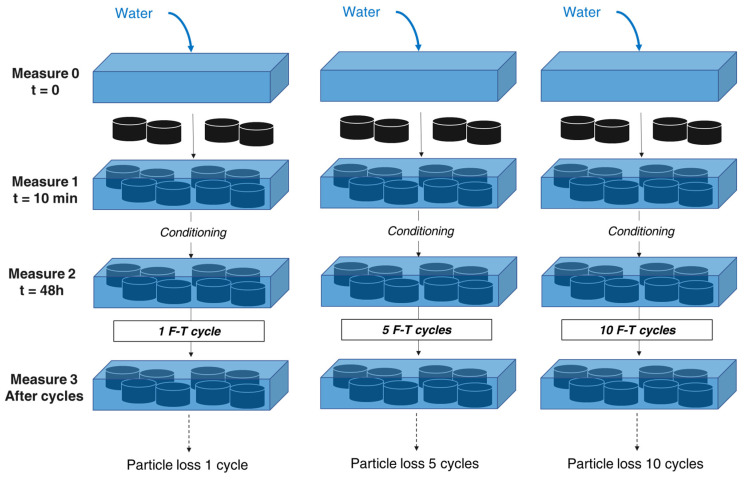
EC-TDS, pH, and particle loss measurement procedure.

These two protocols were conducted to have a quantitative view of the leaching and potential release of the rubberised asphalt developed during the F–T cycles procedure. The complete procedure is illustrated in Figure 4. 

## 3. Results and Discussion

### 3.1. Volumetric, Geometrical Properties of the Mixtures and Relevant Observations

The sample visual aspect is variable because of the type of binder used. The specimens made with the PmE have a softer consistency and a smoother surface texture than those made with PmB, and the specimens made with the PmE are generally softer than the others. Regarding the air voids’ content, as shown in Table 2, the mixes are ranked as follows R1-C > R2-C > R1-W > R2-W. The samples made with PmE expand more and contain more voids than those made with PmB. In the case of the cold binder-based specimens, the expelled water can partially contribute to creating voids. Furthermore, the absence of heat also makes the rubber less malleable and less prone to digestion, i.e., swelling.

**Figure 5:**
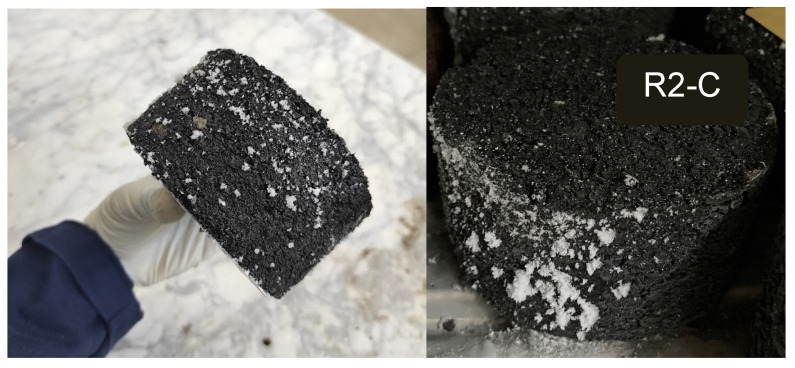
R2-C aspect after the 5th freezing cycle of the F–T cycles.

Any noticeable degradation was not observed on the samples during the F–T procedure. This observation was also made by Richardson et al., acknowledging the potential of the rubber to protect the concrete from F–T [20]. However, ice formation has been observed on the samples made with Rubber 2 and PmE binder (R2-C), but not on the samples made with the other mixes, as shown in Figure 5.

### 3.2. Mechanical Performances

#### 3.2.1. ITSM at 5 °C

**Figure 6:**
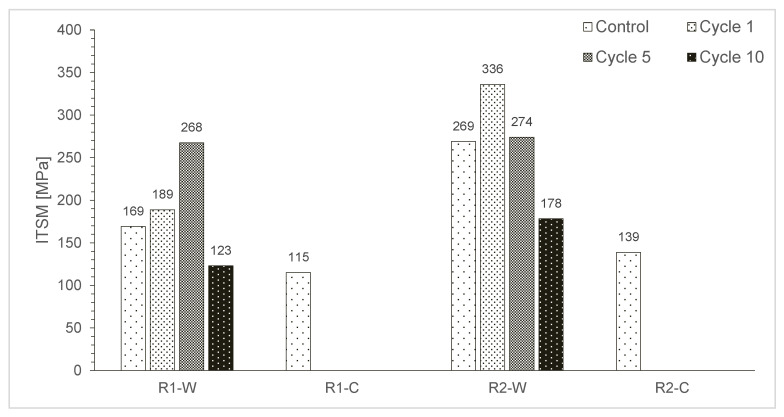
ITSM (@5 °C) values for each mixture vs. cycles.

The ITSMs of the rubberised asphalt specimen were measured at 5 °C for control at t = 0, and after 1, 5, and 10 F–T cycles and are shown in Figure 6. 

At t = 0, the R1-C mixture exhibits the lowest stiffness values, while the R2-W has the highest. The mixture R2-W has a higher ITSM than R1-W, and R2-C records a higher stiffness than R1-C.

The R2-W mixture showed a higher stiffness than R1.W during the F–T cycles. In parallel, the overall tendency for R1-W and R2-W specimens is to increase their stiffness in the middle of the freeze and thaw procedure (precisely between cycles 1 and 5), before considerably decreasing at cycle 10 and reaching lower values than the other those of the control group.

The ageing of the binder can explain the initial increase in stiffness, and this phenomenon occurs in the first part of the procedure. The water diffusion inside of the samples may cause the decrease in the stiffness observed at the end, causing air voids to increase in the matrix, an expansion of the porosity, and confirming a lower ITSM value.

**Figure 7:**
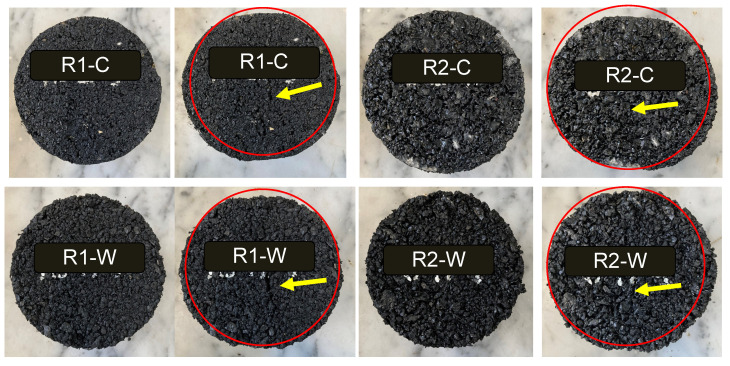
Comparison of the samples after 10 F–T cycles. On the left, the samples before ITS test; on the right, samples after testing. The red circle represents the sample’s deformation, and the arrow highlights the crack visible on the samples.

Figure 7 shows that all samples could be tested at the control time (0 F–T) although sometimes it was challenging. On the contrary, the measurement of ITSM values at 1, 5, and 10 cycles was often impossible, mainly because of the low values and testing device limits.

Generally, R2-W is stiffer than R1-W, and the specimen made with PmE are softer than those made with PmB. This was also macroscopically evident while handling the samples. Thus, it was not possible to record the ITSM evolution for the R1-C and R2-C mixtures. In this case, a uniaxial compressive analysis could be a valid alternative to the ITSM. 

#### 3.2.2. ITS at 5 °C

**Figure 8:**
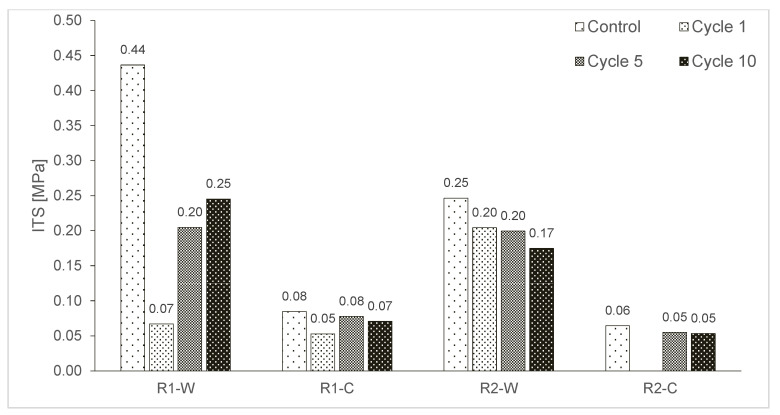
ITS values for tests carried out at 5 °C and different cycles.

The ITS tests of the rubberised asphalt specimens were performed at 5 °C for control at t = 0, and after 1, 5, and 10 F–T cycles. The visual and graphical results are presented in Figure 7 and Figure 8.

In general, as shown in Figure 8, due to the high amount of rubber in the mixture, the cracks developed during testing are hard to see. However, the elastic behaviour of the materials was evident. At failure, samples are still cohesive and tend to spring back to their original shape. 

Graphically, Figure 8 shows that at t = 0, mixture R1-W had higher strength than R2-W, and R1-C had higher strength than R2-C. R1-W gave the highest value at 0.44 MPa, and R2-C the lowest at 0.06 MPa. Samples made with the warm bitumen had higher ITS than those made with cold emulsion. In the warm method, the temperature can contribute to the physico-chemical interactions between the constituents, in particular bitumen and rubber. In the cold mix, rubber is not heated, which allows the drop in fumes and odour emissions, but lacks in creating a strong cohesive matrix and the air voids’ content remains higher, thus reducing the tensile strength of the -C samples.

With the increase in cycles, the ITS of samples made with PmB tends to decrease while the ITS of the samples made PmE does not vary. R1-W registered the highest reduction in strength along with the cycles. The decrease in the ITS registered for the PmB based samples can be explained by the same mechanism observed for the ITSM values.

Generally, the specimens made with PmE have a lower ITS than those made with PmB. The samples made with Rubber 1 have slightly higher ITS than those made with Rubber 2. Regarding the PmE based samples, a change in the visible mechanical structure is not evident. Indeed, the values of the ITS at 0 F–T cycles are low, and the same tendency is observed after all cycles. No increase or decrease in the ITS values is easily observed.

### 3.3. Particle Loss

#### 3.3.1. Cantabro Loss

**Figure 9:**
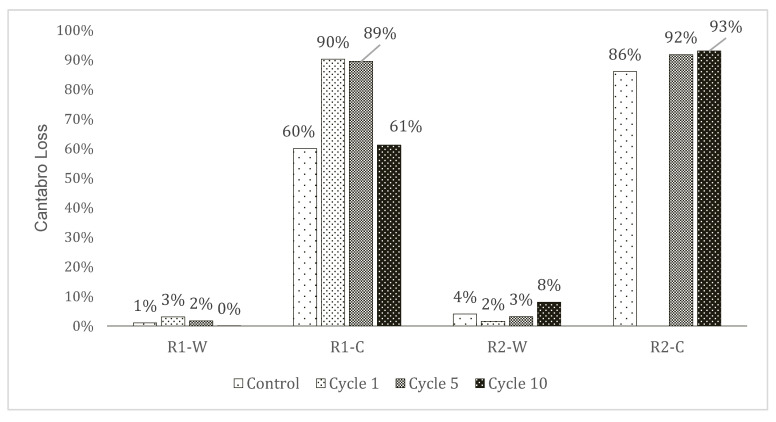
Cantabro loss percentage of the rubberised asphalt specimens at different stages of the F–T procedure.

**Figure 10:**
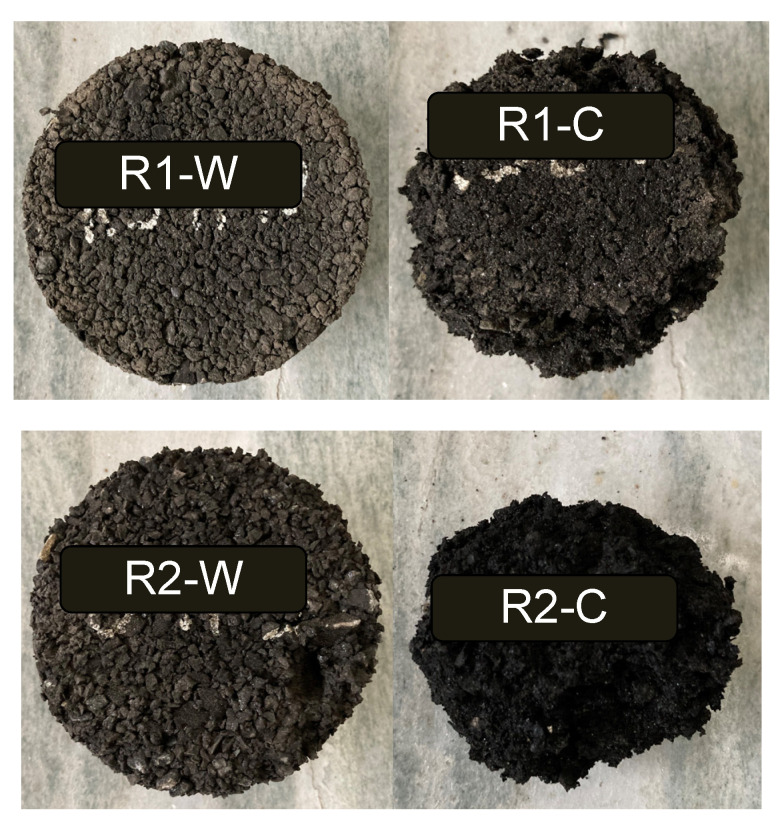
Aspect of the samples after the Cantabro test at the control time (no F–T).

The Cantabro loss of the rubberised asphalt specimens was measured at t = 0 (control), and after 1, 5, and 10 F–T cycles. Results are shown in Figure 9 and Figure 10.

For R1-W samples, the registered loss is stable, varying from 0% to 3% maximum. This range is the same observed in a previous study for a reference mix without rubber and made with SBS-PmB [23]. The Cantabro loss measured was around 1% without any F–T cycles, and a higher loss is observed after cycle 1. Concerning R2-W, the Cantabro loss is doubled comparing the control to cycle 10, with losses from 4% to 8%. Using the same warm binder, the mixture produced with R1 is stronger than with R2. 

R1-C behaviour is hard to explain; the loss at t = 0 is 60%; this value increases to approximately 90% in cycles 1 and 5, and it goes back to 61% after 10 cycles. However, with respect to R2-C, with the same binder, an increase in the amount of loss with cycles is evident. The loss values of R2-C samples at t = 0, 5, and 10 cycles are higher than those of R1-C ones.

As shown in Figure 9 and Figure 10, in the case of R1-W, the F–T cycles seem not to strongly influence the loss. The durability of the mix is very high with reference to the applied F–T procedure. Effects of F–T cycles are visible on R2-W samples. R1-C and R2-C, both made with cold emulsion, are prone to consistent degradation due to the F–T. Nevertheless, the R1-C specimens appear to be somehow stronger. Figure 10 also visually corroborates how the rubberised asphalt concretes made with Rubber 1 showed lower values in terms of the Cantabro loss test. 

#### 3.3.2. Particle Loss in the Thaw Water

**Figure 11:**
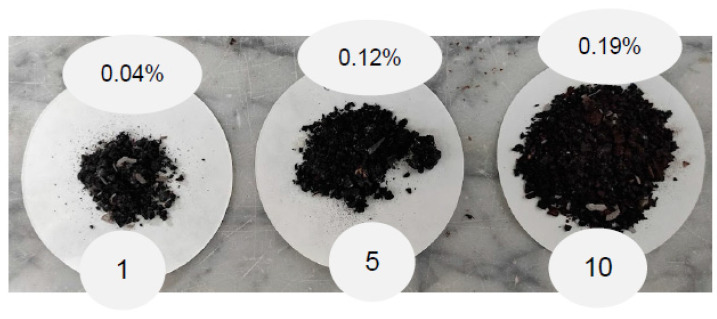
Loss of asphalt concrete samples material in the thaw waters after 1, 5 and 10 F–T cycles.

The particles lost in the thawing water were collected after 1, 5, and 10 cycles of F–T. Figure 11 shows that the alteration caused by the F–T procedure induces the release of particles in the water. In cycle 1, the loss is 0.04%, in cycle 5, it is 0.12% and in cycle 10 the collected material corresponds to 0.19% of the total weight of the samples. This kind of loss in particles is often observed in standard asphalt concretes, where the iterative variation of temperatures can cause premature degradation of the material [2]. The results show that, in parallel to the Cantabro test results, the loss is higher for the mix made with the cold binder, which appeared more degradable. However, the measured loss is not a critical factor, and optimisation of the mix, such as a change in the gradation curve or improved control of the mixing process, could tackle the problem.

### 3.4. Leaching Analysis

**Figure 12:**
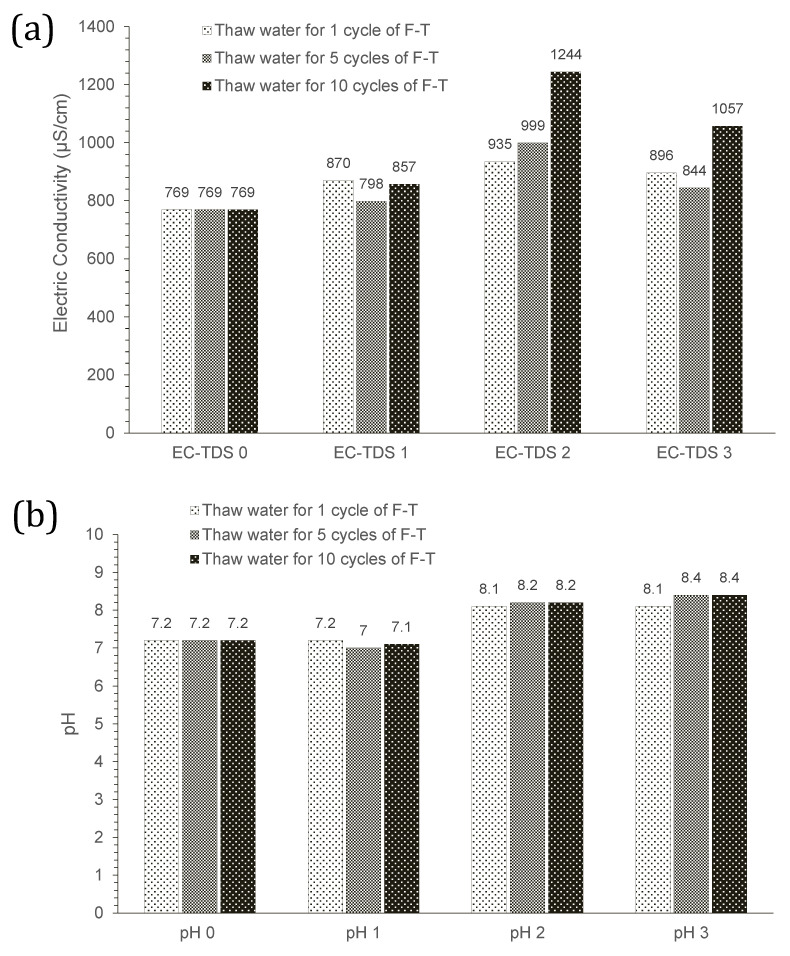
Changes in (**a**) electric conductivity and (**b**) pH of thaw waters at different stages of F–T procedure for rubberised asphalt specimens.

The leaching behaviour of materials is often studied because of its necessity in ensuring the safety of the materials used, in this case, the construction materials’ safety and verifying their non-hazardousness to the environment. The leaching values collection was done by measuring the pH and electric conductivity of the water in contact with the rubberised asphalt samples during the F–T conditioning cycles. Data were collected before starting the F–T cycles at t = 0, t = 10 min, t = 48 h, and during the F–T procedure at the end of the 1st, 5th, and 10th F–T cycles. Results are presented in Figure 12a. The highest average electric conductivities were observed before starting the cycles after the 48 h of conditioning. Values decrease at the end of each cycle group (1, 5, or 10). The ECs after 1 cycle or 5 cycles are relatively similar, while after 10 cycles, the measured EC value increases. This observation revealed that the mixtures released some ions, and a higher number of ions is released in a short time into the water (after only 48 h). The EC also increases in relation to the time of contact of the samples with water.

The pH evolution of water was also tracked for the three thawing waters and is shown in Figure 12b. According to the results, the water pH from the F–T cycles increased as the duration of contact increased. At t = 10 min, the pH of the water was verified, and it was relatively stable compared to the original water pH (7.2). After the 48 h conditioning period, the pH for all waters increased to 8.1–8.2. The pH of the three thaw waters increased from 7.2 to above 8 between day 0 and day 2 and stabilised around 8.1 and 8.4 already after 1 cycle of F–T. However, the maximum pH (8.4) was observed at the last stage when samples were in contact with the water for longer (5 or 10 cycles). This observation confirms the EC results where the higher release of ions was observed after 5 or 10 cycles, regardless of the thaw waters. The maximum pH values for the rubberised materials occurring at earlier stages (after the conditioning period) were also in line with their maximum electric conductivity records at earlier stages (Figure 12a).

The obtained values of EC and pH are comparable to those observed in a parallel study on highly rubberised asphalt leaching. In the study, the EC value increased at an early stage before decreasing, while the pH values were initially found below 7.5 and subsequently increased to 8–8.5 after 4–9 days, corresponding to the approximate time required for the present F–T procedure. It was also demonstrated that for a mix containing 56% rubber made with a cold or warm SBS modified bitumen, the recorded values were comprised in the thresholds set by the Dutch Soil Quality Decree [31]. 

## 4. Conclusions

This paper is part of a wider project aiming to develop a highly rubberised paving material that can offer important impact-absorbing performances and allow, in parallel, the recycling of end-of-life tyres in large quantities. Only a few studies have assessed similar materials in cold climate conditions and their potential degradation after several freeze and thaw cycles. The research investigated the stiffness, strength, and cohesive properties of the newly developed material and it also measured the leaching of mixtures made with two different rubbers (1 and 2), bound with a warm (W) and a cold asphalt binder (C).

- The visual, geometrical, and volumetric assessments have shown that the specimens made with the cold binder are softer, expand more and contain more air voids than other samples.
- The ITSM test outcomes show that R2-W is stiffer than R1-W, and that specimens made with PmE are softer than those made with PmB. The cold mixtures were too soft even at 5 °C and could not be tested with the ITSM procedure. In this case, a uniaxial compressive analysis should be preferred.
- The ITS of specimens made with PmE are lower than those made with PmB. The samples made with Rubber 1 have slightly higher ITS than those made with Rubber 2. Regarding the PmE samples, a modification in the mechanical structure is less evident, certainly because the control values start low and no main fluctuation is observed, while the PmB-made samples show a clear decrease in the ITS value with the F–T cycles.
- Concerning the Cantabro loss method, the variation of particles lost along the F–T cycles is very low for the cold specimens. The mixtures made with Rubber 1 record lower loss values after the Cantabro test and R1-W have similar Cantabro loss than a standard asphalt concrete (without rubber) at 0 cycles.
- The values of EC and pH are comparable to those observed in a parallel study on highly rubberised asphalt leaching The leaching is mainly observed in the first hours. The early leaching observed could be handled with several solutions such as rubber pre-treatment or water washing before their incorporation into the mix to limit and prevent their leaching into the pavement and consequently into the environment.

The cold mix should be preferred because of its important advantage regarding the workability and production methods by reducing externalities. However, improvements must be done to propose a viable and durable solution under freeze–thaw conditions, especially regarding the loss of particles.

Future studies should analyse the F–T effect of specimens made with the same formulation but incorporating rubber from a single source and treated with various surface modification methods.

## Figures and Tables

**Table 1:** Mixture composition in the percentage of the volume of the total mixture.

(Volume of the Total Mix)	Warm PmB (SBS)		Cold PmE (SBS)	
	R1-W	R2-W	R1-C	R2-C
Rubber amount [%]	56		56	
Binder amount [%]	15		18	
Aggregates and filler [%]	29		26	

**Table 2:** Densities and air voids of the produced specimens.

Mixes	EN12697-6 D Density [kg/m^3^]	EN12697-5 B Maximum Density [kg/m^3^]	EN12697-8 VA (%)	EN12697-8 VMA (%)
R1-W	1.467 ± 0.058	1.396	4.7 ± 3.7	34.0 ± 4.8
R2-W	1.406 ± 0.030	1.458	3.5 ± 2.0	31.7 ± 1.4
R1-C	1.300 ± 0.036	1.544	16.6 ± 2.3	42.4 ± 1.3
R2-C	1.430 ± 0.026	1.605	11.1 ± 1.6	39.7 ± 1.1

## Data Availability

Not applicable.

## Appendix A

The appendix contains the materials data and details, including their typical values and properties.

### Appendix A.1. Rubbers

**Table A1:** Crumb rubber typical values.

Rubber	Granulates	Particle Size [mm] ISO 13322-2	Bulk Density [kg/m^3^] EN 1097-3	Specific Density [kg/m^3^]	PAHs 8 REACH [mg/kg] (Specification ≤ 20)
Rubber 1	Fine	0–1.2	0.440	1.028 (EN 1097-6) *	6.5
	Medium	1.0–2.8	0.440	1.028 (EN 1097-6) *	6.5
	Coarse	2.5–4.0	0.440	1.028 (EN 1097-6) *	6.5
Rubber 2	Powder	0.2–0.8	0.345	1.160 (ASTM D1817-05)	<15
	Fine	0.8–3.0	0.420	1.160 (ASTM D1817-05)	<15
	Coarse	2.0–4.0	0.455	1.160 (ASTM D1817-05)	<15

* Experimental value. All the other Typical properties are given by the manufacturers.

**Table A2:** Crumb rubber sieve analysis.

	Percent Retained per Sieve [%]					
	Rubber 1			Rubber 2		
Sieve (mm)	Fine	Medium	Large	Powder	Fine Mix	Coarse
4	0.00	0.00	0.15	0.00	0.00	0.00
2	0.00	34.18	97.90	0.00	22.00	95.79
1	0.09	58.68	1.88	0.00	73.88	4.18
0.5	65.96	6.94	0.00	85.30	4.01	0.04
0.25	23.91	0.02	0.01	14.50	0.03	0.00
0.125	8.22	0.05	0.03	0.20	0.01	0.00
0.063	1.71	0.10	0.03	0.00	0.05	0.00
<0.063	0.11	0.03	0.00	0.00	0.02	0.00
Total	100.00	100.00	100.00	100.00	100.00	100.00

### Appendix A.2. Aggregates

**Table A3:** Aggregates typical values.

Virgin Aggregates	Particle Size [mm] EN 933-1	Specific Density EN 1097-6
Limestone 1	8–14	2.661
Limestone 2	4–8	2.669
Limestone filler	≤0.063	2.667

**Table A4:** Aggregates sieve analysis.

Percent Retained per Sieve [%]		
Sieve (mm)	4–8	8–14
14	0	1.87
12.5	0	5.64
10	0	26.65
8	0.71	29.75
6.3	34.24	30.79
4	56.15	4.85
2	8.83	0.29
1	0.04	0.07
0.5	0.02	0.03
0.25	0.01	0.02
0.125	0.01	0.01
0.063	0.00	0.01
<0.063	0.01	0.04
Total	100.00	100.00

### Appendix A.3. Bitumen

**Table A5:** Typical Bitumen values.

Code	Modifier	Penetration (25 °C)	Softening Point	Resistance to Hardening RTFOT (EN 12607-1)			Flash Point COC	Dynamic Viscosity
				Change in Mass (Absolute Value)	Retained Penetration (25 °C)	Increase in Softening Point (Severity 1)		
		EN 1426	EN 1427	-	EN 1426	EN 1427	EN ISO 2592	EN 13702
		dmm	°C	%	%	°C	°C	mPa.s
W	SBS	25–55	≥70	≤0.5	≥65	≤8	≥250	295 (135 °C)

### Appendix A.4. Emulsion

**Table A6:** Emulsion typical values.

Code	Modifier	Binder Content	Breaking Index	Viscosity at 40 °C (Cup Discharge Time 4 mm)	Adhesiveness	Penetration (25 °C)	Softening Point
		EN 1428	EN 13075-1	EN 12846	EN 13614	EN 1426	EN 1427
		%	-	sec	%	dmm	°C
C	SBS	67–69	70–155	5–70	≥90	45–80	≥60
